# Concomitant acute myocardial infarction and acute pulmonary embolism caused by paradoxical embolism: a case report

**DOI:** 10.1186/s12872-021-02123-1

**Published:** 2021-06-24

**Authors:** Weiwei Chen, Zhixi Yu, Siming Li, Kenji Wagatsuma, Beibei Du, Ping Yang

**Affiliations:** 1 Department of Cardiology, China-Japan Union Hospital of Jilin University, Xiantai Street No. 126, Changchun, 130031 Jilin China; 2 Jilin Provincial Cardiovascular Research Center, Jilin Provincial Engineering Laboratory for Endothelial Function and Genetic Diagnosis of Cardiovascular Disease, Changchun, 130031 China; 3 Tsukuba Heart Center, Tsukuba Memorial Hospital, Tsukuba, Ibaraki, Japan

**Keywords:** Acute myocardial infarction, Paradoxical embolism, Patent foramen ovale, Acute pulmonary embolism, Case report

## Abstract

**Background:**

Due to its low incidence and diverse manifestations, paradoxical embolism (PDE) is still under-reported and is not routinely considered in differential diagnoses. Concomitant acute myocardial infarction (AMI) and acute pulmonary embolism (PE) caused by PDE has rarely been reported.

**Case presentation:**

A 45-year-old woman presented with acute chest pain and difficulty with breathing. Multiple imaging modules including ECG, echocardiography, emergency cardioangiogram (CAG), and CT angiography of the pulmonary arteries showed acute occlusion of the posterolateral artery and acute PE. After coronary aspiration, no residual stenosis was observed. One month later, a bubble study showed inter-atrial communication via a patent foramen ovale (PFO). The AMI in this patient was finally attributed to PDE via the PFO. PFO closure was performed, and long-term anticoagulation was prescribed to prevent recurrent thromboembolic events.

**Conclusions:**

PDE via PFO is a rare etiology of AMI, especially in patients with concomitant AMI and PE. Clinicians should be vigilant of this possibility and close the inter-atrial channel for secondary prevention.

## Background

Paradoxical embolism (PDE) was first described by the German pathologists Cohnheim et al., who found a large number of emboli in the cerebral artery in an autopsy patient, and at the same time, a long embolus in the femoral vein. PDE refers to a embolus from the vein or right heart system that passes through the right to left shunt to the left heart, resulting in systemic embolism [1]. Patent foramen ovale (PFO) is one of the inter-atrial channels that causes PDE. The brain is the organ most commonly affected by PDE, resulting in cryptogenic stroke or transient ischemic attack (TIA), followed by the limbs and internal organs, and involvement of the coronary arteries is rarely reported [2]. For management after the diagnosis of PDE, closure of PFO was recommended in selected patients to prevent further systemic embolism [3].

Acute myocardial infarction (AMI) and acute pulmonary embolism (PE) are both life-threatening diseases that initially present with acute chest pain. Clinicians should be vigilant of the possibility of coronary embolism in the differential diagnosis of acute chest pain in young patients with AMI. When acute PE exists, the acutely increased right atrial pressure reopens the closed PFO and facilitates PDE to the brain or coronary artery [4]. Here, we report the diagnosis and management of a young patient with AMI caused by PDE, and acute PE.

## Case presentation

**Fig. 1:**
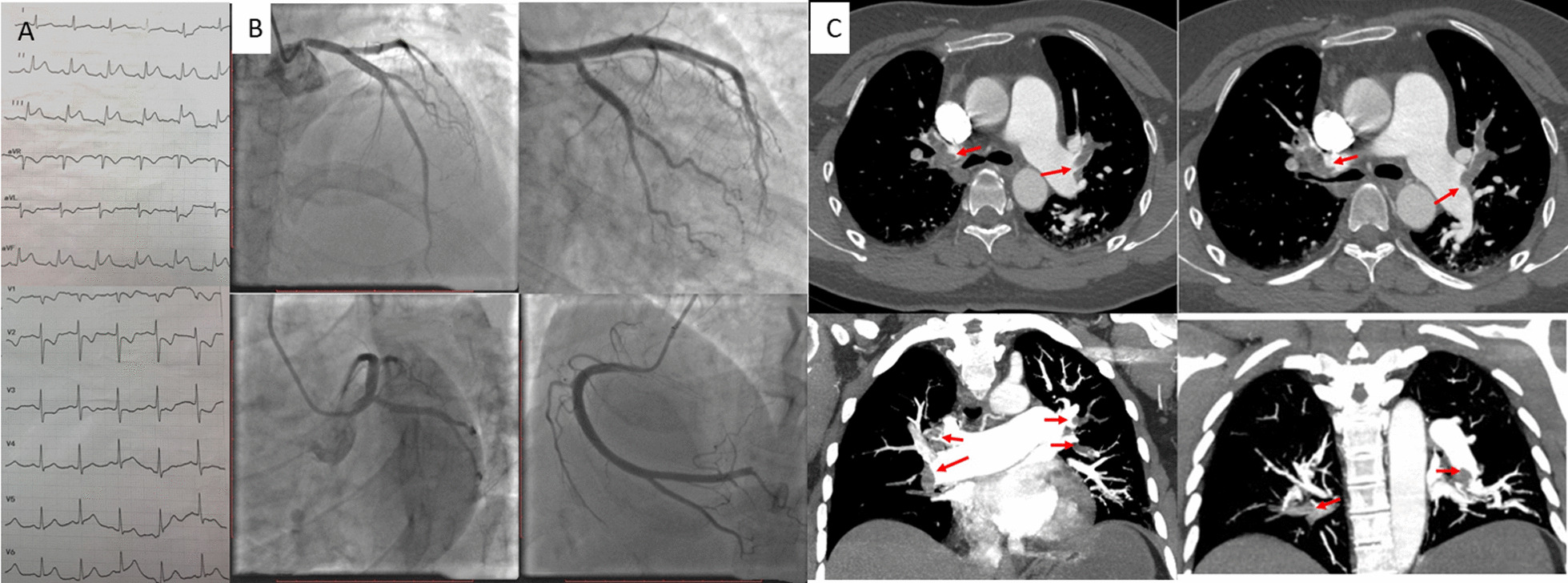
Admission ECG, pulmonary CTA and CAG findings. Admission ECG showed ST elevation in the inferior wall leads (**A**), emergency CAG showed acute occlusion of the PL (**B**), and admission pulmonary CTA showed multiple emboli (red arrows) in the pulmonary arteries and branches (**C**). CAG, cardioangiogram; PL, posterolateral artery; CTA, CT angiography

A 45-year-old female patient was admitted to our hospital with acute chest pain and difficulty in breathing for 2 h. Her past medical history was notable for an orthopedic surgical procedure and a confirmed pulmonary embolism, which was managed with thrombolytic therapy in the acute phase and oral anticoagulation treatment (rivaroxaban 15 mg bid for 1 month; 20 mg qd thereafter). Physical examination showed normal BP (110/64 mmHg) and rales in the lungs. On admission, she underwent an ECG (Fig. 1A), which showed ST elevation in the inferior wall leads. Bedside echocardiography demonstrated inferior wall hypokinesis and mildly increased pulmonary artery pressure (35 mmHg), with normal cardiac function (EF, 63%). Cardiac injury biomarkers were as follows: cardiac troponin I (cTnI) 0.06 ng/ml ↑, myoglobin 12.0 ug/ml ↑. Other tests showed elevated D-dimers level (2910 ng/mL ↑), and blood gas test showed decreased PO_2_ (55 mmHg) and normal PCO_2_ (26 mmHg). An emergency cardioangiogram (CAG) revealed acute occlusion of the posterolateral artery (PL) (Fig. 1B), and CTA of the pulmonary arteries revealed acute pulmonary embolism (Fig. 1C, red arrows).

Based on the symptoms, past medical history, CAG, and pulmonary CTA findings, the patient was diagnosed with acute inferior wall STEMI, Killip class I, acute pulmonary embolism (intermediate-risk group), and type I respiratory failure.

**Fig. 2:**
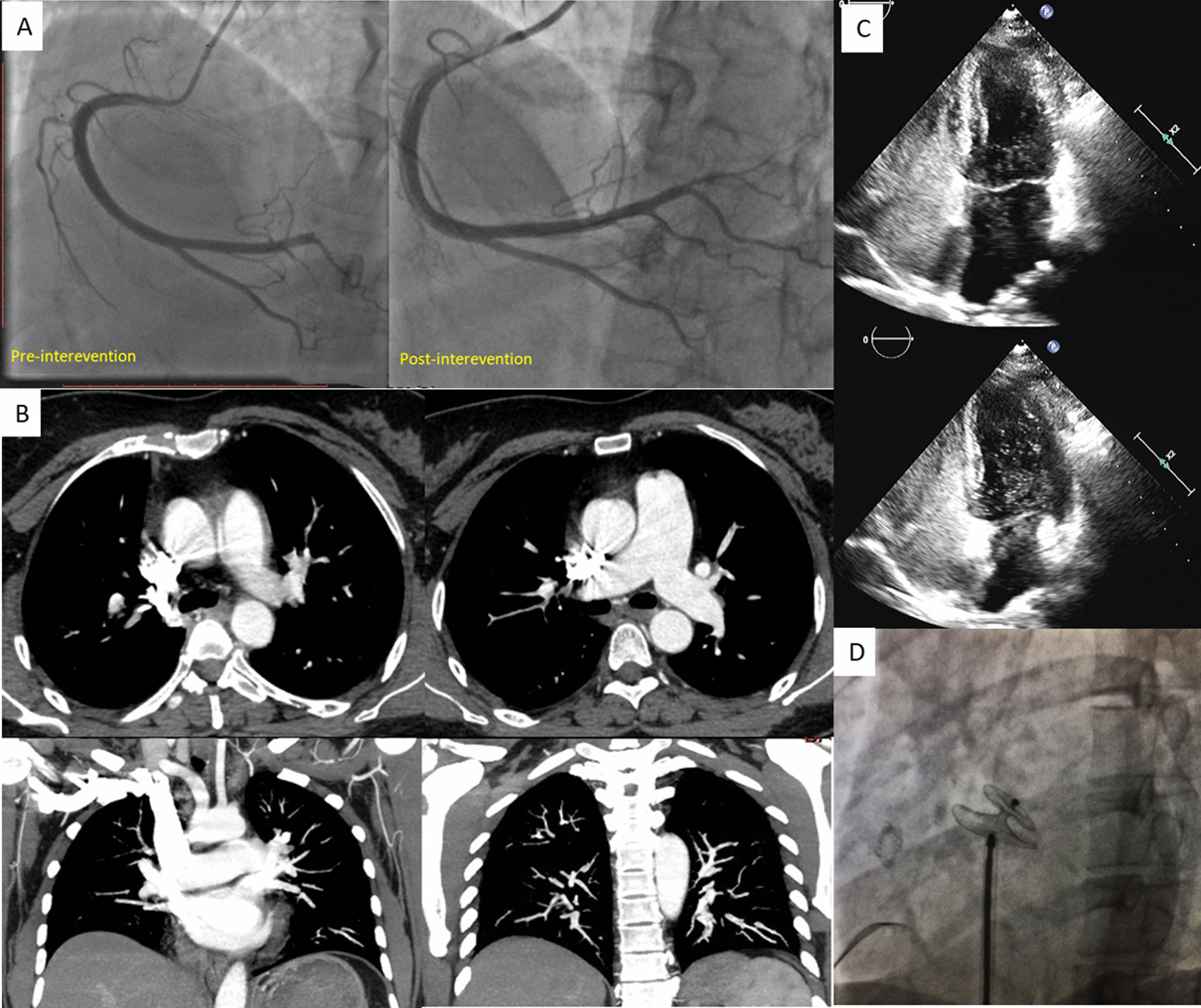
Procedure, post-procedure findings and management. **A** Post-intervention CAG showed no residual stenosis after embolus aspiration. **B** Pulmonary CTA at the one-month follow-up showed no residual emboli. **C** Bubble study showed > 10 bubbles crossed into LA in 1 cardiac cycle (upper: bubbles in LA and LV; lower: bubbles in LV). **D** PFO closure of this patient. CAG, cardioangiogram; CTA, CT angiography; PFO, patent foramen ovale

In consideration of the need for coronary intervention, after embolus aspiration, coronary flow returned to TIMI grade 3, and no residual stenosis or plaque remained (Fig. 2A). Intracoronary imaging was recommended to confirm the cause of the acute occlusion, but it was declined by the patient. No balloon dilation or stent implantation was performed.

The relationship between AMI and PE in this patient is unknown. On lower extremity vascular ultrasound, rheumatic disease-related embolism and tumor-related emboli were ruled out. Because of the concern engendered by the increase in pulmonary artery pressure, a bubble study was not recommended (owing to the risk of further increase in pulmonary artery pressure) by the echocardiologist, and paradoxical embolism could not be ruled out.

Triple antithrombotic therapy was recommended (aspirin, clopidogrel, and factor Xa inhibitor for one month; clopidogrel and factor Xa inhibitor until one year; factor Xa inhibitor lifelong). At the one-month follow-up, pulmonary CTA showed no residual embolism (Fig. 2C). Echocardiography revealed normal pulmonary artery pressure. A bubble study showed > 10 bubbles crossing into the LA in one cardiac cycle (Fig. 2^2^), which was considered an echocardiographic feature of paradoxical embolism [5]. Paradoxical embolism via the interatrial channel was considered the etiology of the chest pain, and patent foramen ovale (PFO) closure was performed for this patient (Fig. 2D). Additionally, the antithrombotic strategy was changed to an anticoagulant (factor Xa inhibitor) only. Three-month and 6-month telephone follow-ups showed good recovery.

## Discussion

Coronary embolism should be considered in patients with AMI, especially in patients with hypercoagulable status [6]. Due to the diverse clinical manifestations, PDE is still under-diagnosed [1]. In particular, AMI caused by PDE is rarely reported, and no large study on this special patient cohort has been conducted.

The diagnosis of PDE is mostly presumptive, except for those with evidence of emboli in-transit (called impending PDE) via PFO [7]. There are three required conditions in the diagnosis: emboli from a venous source, abnormal inter-cardiac communication (atrial septal defect or PFO), and evidence of systemic embolism [1, 8]. In this case, the patient met the three conditions, and the source of the embolism in PL was not the left ventricle or other arteries. Coronary embolism from other sources was excluded.

AMI and acute PE are both life-threatening diseases presenting with acute chest pain. Concomitant AMI and acute PE have been reported in a limited number of cases [3, 9, 10] and interestingly, PDE is the common etiology of coronary embolism. Acute PE seems to facilitate the travel of embolism via PFO to the left heart system [18]. In a prospective observational study of 139 patients with major PE, patients were at a significantly higher risk of stroke and peripheral arterial embolism in the PFO group than in patients without PFO [18]. From a pathophysiological perspective, acute PE increases right heart pressure, causes the foramen ovale to reopen, and subsequently facilitates PDE [4]. In this case, the bubble study also verified the right-to-left shunt via PFO after acute PE.

Concomitant AMI and PE are indicative of a PDE. However, the diagnosis of concomitant AMI and PE is sometimes challenging (Table 1) because the two conditions share the similar symptom of acute chest pain. For this special clinical scenario, a past history of deep vein thrombosis (DVT) or PE, or accompanying symptoms (difficulty in breathing, cough, unexplained dyspnea, or respiratory failure) is helpful for clinicians to differentiate between AMI and PE. In this case, the diagnosis was prompted by the past medical history and the accompanying symptoms.

**Table 1:** PDE cases reported in MEDLINE/PubMed and scopus databases

No.	Author [Ref.]	Biographical information	Diagnosis	Treatment (Coronary; Pulmonary and PFO closure)
1	Collado Fareed Moses et al. [3]	71 years female	NSTEMI; PE; PFO	C: Occlusion of RCA; Aspiration thrombectomy; Antiplatelet treatment not mentioned Pul: Not mentioned PFO closure: Not suitable due to severe pulmonary hypertension
2	Rovner et al. [11]	70 years female	STEMI (anterolateral wall); PE; PFO	C: No acute occlusion; Lucent area in OM; Antiplatelet treatment not mentioned Pul: Oral anticoagulant (Warfarin) PFO closure: Deferred due to sepsis from a urinary tract infection; Later lost to follow-up
3	Hline et al. [12]	86 years female	STEMI (inferior wall); PE; PFO	C: Occlusion of RCA. Aspiration thrombectomy; Intracoronary heparin and abciximab; Antiplatelet treatment not mentioned Pul: Lifelong anticoagulant (Warfarin) PFO closure: Multidisciplinary discussion not recommend
4	Smith et al. [10]	69 years female	STEMI (inferior wall); PE; PFO; Acute RV Failure	C: Occlusion of PD and PL; Aspiration thrombectomy and balloon dilation; Oral aspirin Pul: Pulmonary angiography, interventional clot fragment and aspiration; and intra-arterial thrombolysis; Long-term anticoagulant (Warfarin) PFO closure: No
5	Knobloch et al. [13]	38 years male	STEMI (inferior wall); PE and DVT. Embolic occlusion of the left popliteal artery and left carotid artery; PFO	C: Normal coronary angiogram Pul: Not mentioned PFO closure: Yes, transcatheter Others: Left popliteal: Embolus removal with Fogarty catheter; Left carotid embolus: surgery
6	Haghi et al. [14]	61 years female	NSTEMI; PE; PFO	C: Balloon angioplasty (OM) and oral Aspirin Pul: Long-term anticoagulant (Warfarin) PFO closure: No
7	Falcetta et al. [15]	68 years male	STEMI (inferior wall); PE and DVT; PFO.	C: Aspiration thrombectomy (RCA) Pul: Surgical removal of embolus in pulmonary trunk; Long-term anticoagulant (Specific drug not mentioned) PFO closure: Surgery removal of the worm-shaped embolus (13 cm) and PFO surgical sutured
8	Uchida et al. [16]	59 yrs male	STEMI (inferior wall); PE; PFO; AF.	C: Intravenous urokinase; Total occlusion of PD; PTCA of PD; Oral Aspirin Pul: Long-term anticoagulant (Warfarin) PFO closure: No
9	Cvetković et al. [17]	75 yrs female	STEMI (posterior wall); Autopsy found PE	C: Total occlusion of RCA. Died during CAG Pul: NA PFO closure: NA

PDE: Paradoxical Embolism; NSTEMI, Non-ST Segment Elevation Myocardial Infarction; STEMI, ST Elevation Myocardial infarction; PE, Pulmonary Embolism; PFO, Patent Foramen Ovale; DVT, Deep Vein Thrombosis; AF: atrial fibrillation; C: coronary; Pul: pulomonary; CAG: coronary angiogram; OM: Obtuse marginal artery; PD: posterior desending artery; PL: posterolateral artery; PTCA: percutaneous transluminal balloon angioplasty; Ref: reference

To confirm the right-to-left shunt via PFO, bubble studies (bubbles seen in the left atrium in less than three cardiac cycles) [19], and transesophageal echocardiography are usually recommended [1]. Other imaging techniques such as multi-slice computed tomography or MRI (for screening emboli -in-transit) and transcranial Doppler sonography (more specific for PDE-related stroke) can also help in the diagnosis of PDE [1].

The treatments for PDE patients vary vastly depending on the size of the embolus and the thromboembolism sites (Table 1). For the treatment of concomitant AMI and PE caused by PDE, the top priority is to evaluate the hemodynamic status and start thrombolysis if the patient is hemodynamically unstable [9]. Although embolus aspiration is not routinely recommended in AMI intervention, it is mandatory in AMI caused by PDE to eliminate the embolus and restore coronary flow. Percutaneous closure of PFO was recommended in patients aged 18 to 65 years with a highly presumed PFO-related systemic embolism [20] for prevention of a second embolic episode. Moreover, anticoagulation is recommended to prevent emboli from the venous system [20].

## Conclusions

PDE via PFO is a rare etiology of AMI, especially in patients with concomitant AMI and PE. Clinicians should be vigilant of this possibility and close the inter-atrial channel for secondary prevention.

## Data Availability

The data/figures used and/or analyzed in this case are available from the corresponding author upon reasonable request.

## Abbreviations

AMI: Acute myocardial infarction
PDE: Paradoxical embolism
PE: Pulmonary embolism
PFO: Patent foramen ovale
TIA: Transient ischemic attack
